# Modified double patch repair with infarct exclusion technique for ventricular septal perforation: a case study

**DOI:** 10.1186/s13019-018-0708-7

**Published:** 2018-01-30

**Authors:** Takuma Yamasaki, Shuhei Fujita, Yuji Kaku, Junko Katagiri, Takeshi Hiramatsu

**Affiliations:** Department of Cardiovascular Surgery, Japanese Red Cross Kyoto Daini Hospital, Kamanza-Dori, Marutamachi-Agaru, Kamigyo-Ku, Kyoto, 602-8026 Japan

**Keywords:** Acute myocardial infarction, Ventricular septal perforation, Double patch repair, Infarct exclusion

## Abstract

**Background:**

Ventricular septal perforation (VSP) after acute myocardial infarction (AMI) is accompanied by the worsening of rapid hemodynamics, resulting in a poor prognosis. In our department, infarct lesions are preoperatively detected with electrocardiogram (ECG)-synchronized contrast computed tomography, and the scope of approach and exclusion is determined. Furthermore, to effectively prevent a residual shunt, modified double patch repair and infarct exclusion techniques were used in combination to preserve left ventricular (LV) function. This method is reported because it considers both techniques as a surgical procedure that can be accomplished relatively easily and simultaneously.

**Case presentation:**

We targeted two consecutive VSP patients who underwent this procedure. It took an average of 1 day from the onset of VSP to surgery. We performed double patch and infarct exclusion for VSP using bovine pericardium via an LV incision. Two patches were marked with a skin pen to anastomose eight mattresses equally. In addition, a one piece-coupled patch was made for infarct exclusion. The two patients were extubated on the day after surgery and intra-aortic balloon pump assistance was also withdrawn. Without perioperative complications, they could leave the intensive care unit after 6.5 days on average. Early postoperative ECG and magnetic resonance angiography showed good LV wall contraction, except at the infarcted area, with no evidence of a residual shunt.

**Conclusion:**

The modified double patch repair with infarct exclusion technique is more effective for preventing a residual shunt and maintaining postoperative cardiac function than either of the techniques alone.

## Background

Ventricular septal perforation (VSP) after acute myocardial infarction (AMI) is accompanied by the worsening of rapid hemodynamics, resulting in a poor prognosis. Arnaoutakis et al. reported on the largest and most recent database of The Society of Thoracic Surgeons in 2012 [1]; operative mortality was found to be 54.1% if repair was attempted within 7 days of AMI. On the other hand, Lundblad et al. reported that the 30-day mortality rate of VSP closure using the infarct exclusion technique was 16.7%, which was significantly better than that of patch closure [2]. Caimmi et al. reported that the 30-day mortality rate of VSP closure using the double patch technique was 18.8%, and it showed good results when no postoperative residual shunt was observed [3]. However, there are no established views on the approaches and surgical techniques because VSP is a rare disease [4]. In our department, infarct lesions are preoperatively detected with electrocardiogram (ECG)-synchronized contrast computed tomography (CT) and the scope of the approach and exclusion is determined. Furthermore, in order to prevent a residual shunt, the modified double patch repair and the infarct exclusion techniques were used in combination to preserve left ventricular (LV) function. We report on our experiences of this technique and review the existing literature.

## Case presentation

We targeted two consecutive VSP patients who underwent this procedure from September to December 2015. Case 1 was a 71-year-old man with AMI onset 7 days prior. The diameter of VSP was 12 mm, the responsible lesion occurred in the left anterior descending artery (LAD) #7–99% delay, the pulmonary blood flow/systemic blood flow (Qp/Qs) was 3.6, and the pulmonary capillary wedge pressure (PCWP) was 22 mmHg. Emergency surgery was performed under artificial respiration management.

Case 2 was a 78-year-old woman with AMI onset 3 days prior. The diameter of VSP was 18 mm, the responsible lesion occurred in the LAD #7-total, the Qp/Q s was 2.6, and PCWP was 20 mmHg. Emergency surgery was performed under intra-aortic balloon pumping (IABP).

**Fig. 1:**
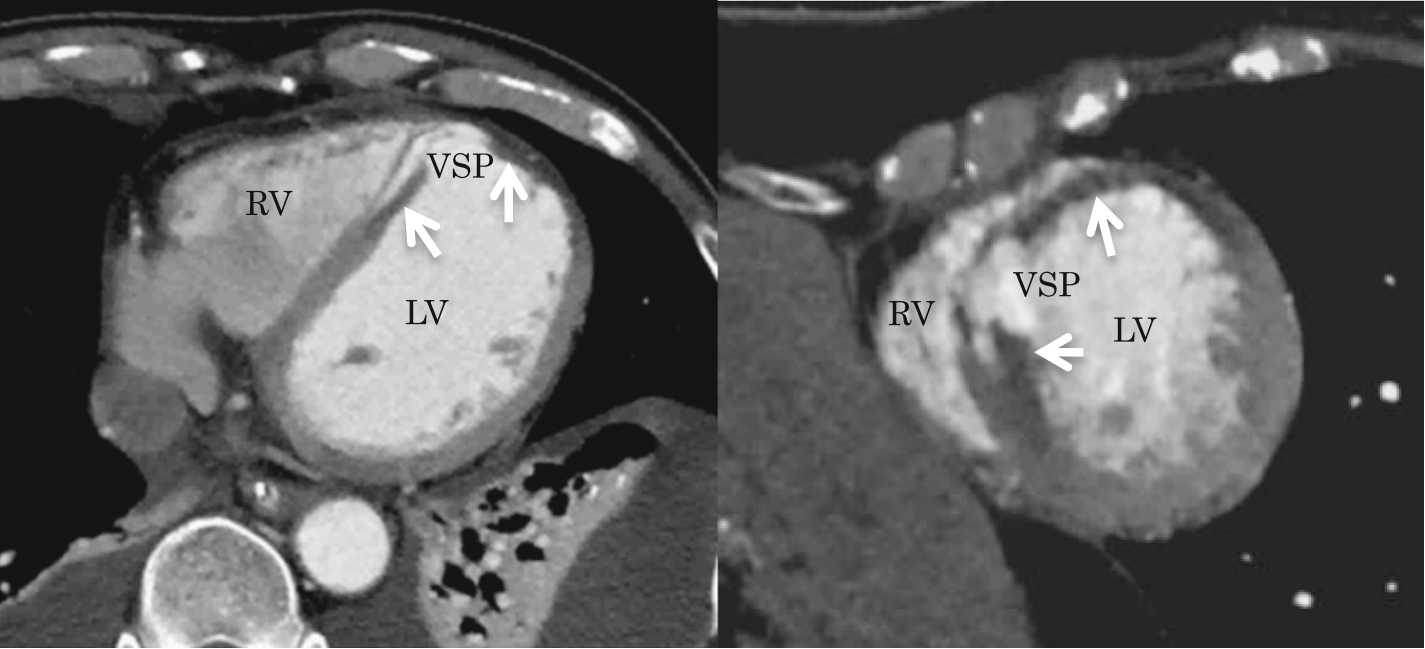
Preoperative computed tomography shows the myocardium having poor contrasting that became light thickness (white arrows)

**Fig. 2:**
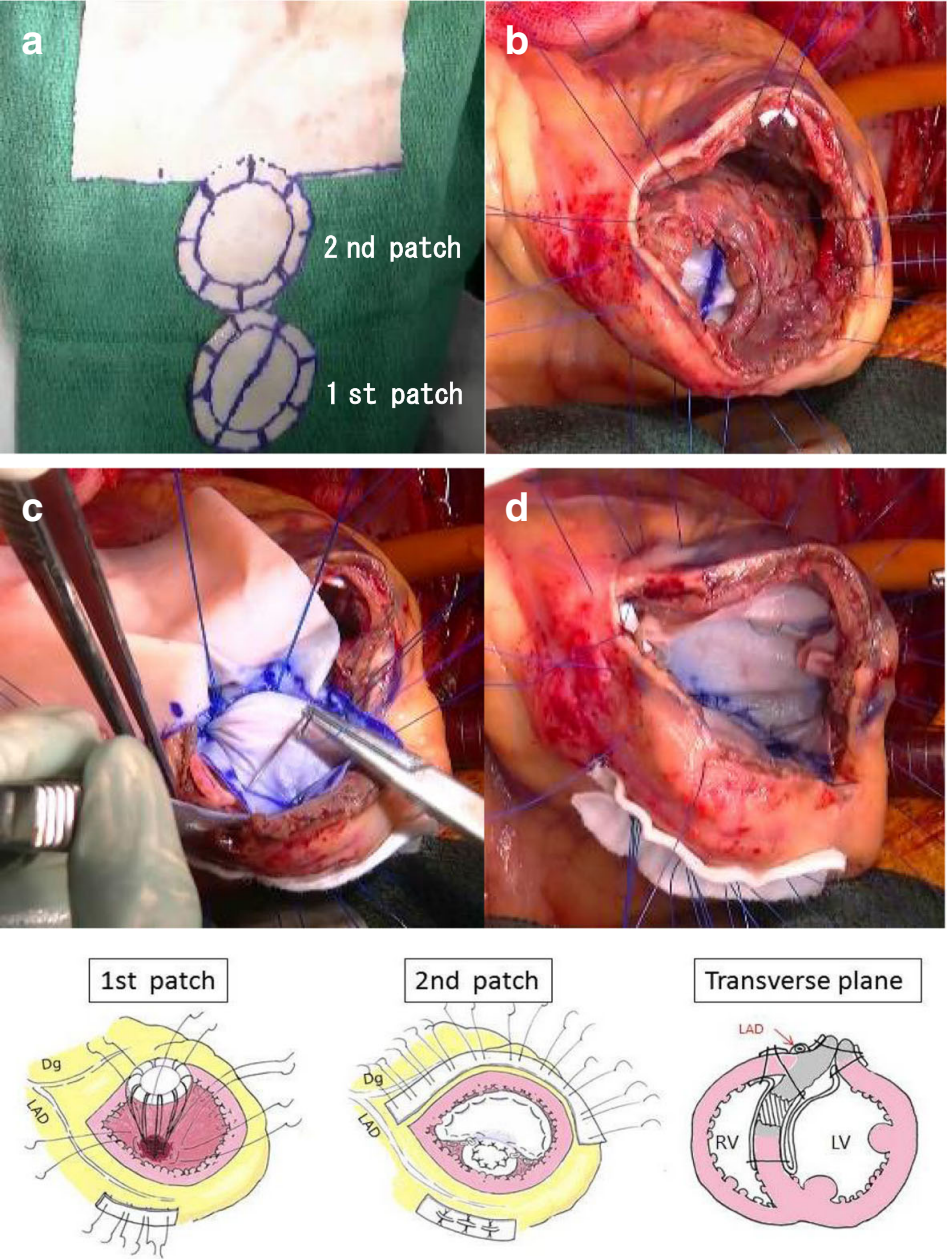
**a** Two patches were made in order to sandwich the septum evenly with an 8-needle mattress. **b** The 1st patch led to the right ventricle via VSP. **c** A set of needle threads penetrating the ventricular septum was passed through the 2nd patch. **d** The patch was appropriately trimmed to exclude the infarcted muscle from the left ventricular cavity. **e** Schema of the modified double patch repair with infarct exclusion technique

**Fig. 3:**
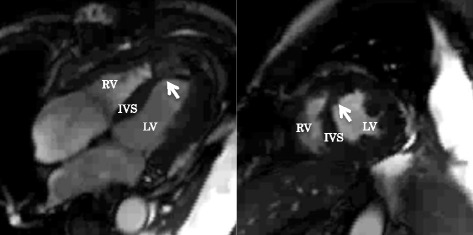
Postoperative magnetic resonance angiography shows the double patch with infarct exclusion (arrow) without residual shunt

ECG-synchronized contrast CT was performed before surgery to identify the infarct area and to set up a surgical strategy (Fig. 1). A median sternotomy incision approach was employed, establishing extracorporeal circulation by ascending aorta and right atrial cannulation. A longitudinal transinfarction incision was performed in the LV myocardium parallel to and 1.5 cm away from the interventricular septum while the heart was beating, and the location of the septal defect was identified. After grasping the boundary between the normal myocardium and the infarcted myocardium manually, antegrade cold blood cardioplegia was infused to arrest the heart. The fragile myocardium surrounding the VSP was excised. A bovine pericardium patch (10 × 15 cm) was trimmed to make a perfect circle with a diameter of 4.5 to 5.5 cm to be used as the 1st patch. Two pieces of patch marked with a skin pen were made to sandwich the septum evenly with an 8-needle mattress. The 2nd patch on the LV side was combined with a patch for infarct exclusion (Fig. 2a). 3–0 polypropylene sutures were concentrically conducted on the 1st patch on the right ventricular (RV) side with an 8-needle mattress; the needle thread was penetrated from the RV side to the LV side into the relatively healthy septal muscle around the VSP. Three needles on the upper edge of the circular patch were inserted into the RV free wall. The 1st patch led to the RV via the VSP (Fig. 2b). A set of needle threads penetrating the ventricular septum were passed through the 2nd patch and gelatin-resorcin-formalin (GRF) glue (Cardial, Technopole, Sainte-tienne, France) or BioGlue (Cryolife Inc., Kennesaw, Georgia, USA) was injected between patches. After completion of the double patch (Fig. 2c), the patch for infarct exclusion was threaded with mattress sutures clockwise from the lower edge of the circular patch. The patch was appropriately trimmed so as not to apply tension to the patch and to exclude infarcted muscle and the double patch from the LV cavity (Fig. 2d). The LV incision line was double suture-closed with the felt sandwich method by using 3–0 polypropylene sutures. Finally, a coronary bypass anastomosis was added to the LAD and the operation was completed (Fig. 2e). In both cases, the anterior LV wall approach was used on the infarcted myocardium side, and coronary artery bypass grafting (CABG) was performed. The average operation time was 290 min, the average aortic cross clamp time was 115 min, and the average extracorporeal circulation time was 192 min. Both patients were extubated on the day after surgery and IABP assistance was also withdrawn. Without perioperative complications, these patients could leave the intensive care unit after 6.5 days and be discharged from hospital after 33.5 days on average. Early postoperative ECG and magnetic resonance angiography showed good LV wall contraction, except at the infarcted area, with no evidence of a residual shunt (Fig. 3), and coronary bypass graft was also patent on coronary artery CT. No symptoms of heart failure occurred, and the patients were discharged without complications.

## Discussion

Acute phase VSP is often difficult to treat surgically because of the unstable circulatory dynamics and fragile myocardium around the VSP. In this report, an intra-aortic balloon pumping was inserted before surgery in one case, and artificial respiration management was needed in the other case as the respiratory and circulatory dynamics had deteriorated; surgery in the acute phase was then judged to be necessary. In this study, we approached from the anterior wall of the LV, the side of the infarcted myocardium, in order to minimize the damage to the non-infarcted myocardium. In addition, to reliably prevent the remaining shunt and maintain cardiac function after surgery, the VSP was closed in both cases by using the modified double patch repair with infarct exclusion technique. This technique can simultaneously achieve the advantages of both the double patch technique and the infarct exclusion technique; therefore, it is considered a useful and effective technique.

Surgical treatment for VSP was greatly improved by the infarct exclusion method reported by Komeda and David et al. They claimed that their technique, which does not resect any part of the RV, could be beneficial in reducing the risk of further RV dysfunction, and that it improved surgical results [5]. However, the hand movement to the LV outflow tract and the LV rear wall at the time of sewing the patch requires considerable ingenuity and skill because the operative field is deep and the posterior papillary muscle is present. As a result, a postoperative shunt remains, requiring reoperation, and results in death from heart failure in many cases [6].

On the other hand, many good results of the double patch method, in which VSP is sandwiched between two patches, have been reported. The advantages of the double patch method for VSP are that it is easy to approach the ventricular lumen from one side and that the procedure can be easily performed. In this method, two patches are uniformly sandwiched between eight needles of mattress nodules so as to sandwich the infarcted septal muscle from the LV and RV; the LV pressure is diffused, thus suturing disintegration can be reduced [7]. In addition, it is possible to increasingly prevent a residual shunt by compensating with glue paste, such as GRF and BioGlue, between patches [8]. Also, in the long term, the double patch method has been reported to alleviate wall motion abnormalities in the ventricular septum [3]. Hosoba et al. reported mid-term results of the extended sandwich patch technique through right ventriculotomy and achieved good results when neither severe septal dyskinesia nor aneurysmal change in the LV was observed [9]. However, in the double patch method based on the RV approach to VSP caused by myocardial infarction in the LAD region, right heart failure due to RV incision may be a problem after surgery [10]. Therefore, we believe this to be a novel procedure as it prevented the residual shunt more effectively by decreasing the LV pressure to the double patch, controlled bleeding from the LV incision line, and prevented postoperative LV aneurysm and rupture. Furthermore, identification of the infarct range with preoperative CT and infarct exclusion with a correctly sized patch can prevent LV remodeling at the remote stage and maintain LV function. By using a 2nd patch as a series of patches, it is relatively easy to perform both the double patch method and infarct exclusion method. However, if the preoperative condition is more imminent, it is difficult to image the CT and identify the infarct range, and similarly, it is considered to be difficult in the case of a more extensive infarction or posterior VSP.

## Conclusion

This modified double patch repair with infarct exclusion technique is more effective than either technique alone for preventing a residual shunt and bleeding of the incision sutures, and can be effective for maintaining postoperative cardiac function.

## Abbreviations

AMI: Acute myocardial infarction
CABG: Coronary artery bypass grafting
CT: Computed tomography
ECG: Electrocardiogram
GRF: Gelatin-resorcin-formalin
IABP: Intra-aortic balloon pumping
ICU: Intensive care unit
LAD: Left anterior descending artery
LV: Left ventricle
PCWP: Pulmonary capillary wedge pressure
Qp/Qs: Pulmonary blood flow/systemic blood flow
RV: Right ventricle
VSP: Ventricular septal perforation
